# Supplementary material for: Short versus long cephalomedullary nails for intertrochanteric femur fractures: A meta-analysis of randomized controlled trials
Source: PLoS One. 2025 May 5;20(5):e0319758. doi: 10.1371/journal.pone.0319758 (PMC12052151; doi:10.1371/journal.pone.0319758)
Supplement: S1 File — (DOCX) [file pone.0319758.s003.docx]

| Author & Year | Random Sequence Generation | Allocation Concealment | Blinding of Participants and Personnel | Blinding of Outcome Assessment | Incomplete Outcome Data | Selective Reporting | Other Bias |
| --- | --- | --- | --- | --- | --- | --- | --- |
| Dragosloveanu et al. | L | L | H | L | L | L | L |
| Garín et al. | L | L | H | U | L | L | L |
| Galanopoulos et al. | L | L | H | L | L | L | L |
| Okcu et al. | L | L | H | L | L | L | L |
| Sahu et al. | L | L | H | U | L | L | L |
| Sellan et al. | L | L | L | L | L | L | L |
| Shannon et al. | L | L | H | L | L | L | L |

U unclear risk; L low risk; H high risk.

Criteria for Each Domain:

1.Random Sequence Generation:

Low Risk: Methods such as random number tables, coin toss, dice roll, computer-generated random numbers, or drawing lots.

High Risk: Quasi-random methods, e.g., allocation based on admission date, birth date, or patient ID number; allocation by personal choice or availability of test results.

Unclear Risk: Mentions "random allocation" or "random" without specific description.

2.Allocation Concealment:

Low Risk: Neither the investigator nor the participants know the upcoming allocation, using techniques like central randomization, coded containers, sequentially numbered, sealed, opaque envelopes, or on-site computer randomization.

High Risk: The investigator or participants are aware of the allocation, e.g., using alternation, or allocation based on date of birth, admission number; unsealed or transparent envelopes.

Unclear Risk: Mentions "random" or "random allocation" but no details on concealment.

3.Blinding of Participants and Personnel:

Low Risk: Blinding applied to main researchers and participants, or no blinding but outcome unlikely to be influenced by lack of it.

High Risk: No blinding.

Unclear Risk: Insufficient information; blinding not mentioned or unclear if it was adequate.

4.Blinding of Outcome Assessment:

Low Risk: Blinding applied to outcome assessors, or no blinding but outcome unlikely to be influenced by lack of it.

High Risk: No blinding.

Unclear Risk: Insufficient information; blinding not mentioned or unclear if it was adequate.

5.Incomplete Outcome Data:

Low Risk: No missing data, or missing data does not significantly affect the results (e.g., few missing patients).

High Risk: Significant missing data, likely to impact effect size (results inconsistent with most similar studies); discrepancies between Per Protocol (PP) and Intention to Treat (ITT) analysis.

Unclear Risk: Incomplete data, or description of data completeness is missing or difficult to assess.

6.Selective Reporting:

Low Risk: Protocol available, all pre-specified outcomes reported. No protocol but all major outcomes reported.

High Risk: Pre-specified major outcomes not fully reported, or outcome measurement/analysis methods not specified in the registered protocol.

Unclear Risk: Insufficient information, difficult to judge if selective reporting is present.

7.Other Sources of Bias:

Low Risk: No significant sources of bias identified in the included studies.

High Risk: Presence of one or more significant biases, e.g., significant baseline imbalances, early study termination.

Unclear Risk: Insufficient information, unclear whether identified issues lead to bias.
