# Supplementary material for: Short versus long cephalomedullary nails for intertrochanteric femur fractures: A meta-analysis of randomized controlled trials
Source: PLoS One. 2025 May 5;20(5):e0319758. doi: 10.1371/journal.pone.0319758 (PMC12052151; doi:10.1371/journal.pone.0319758)

Supplementary Table 1 Search strategy in PubMed, Embase, and Web of Science.

| Database | Search strategy |
| --- | --- |
| PubMed(1666) | ("Fracture Fixation, Intramedullary"[Mesh] OR “Intramedullary Nailing”[Title/Abstract] OR “Intramedullary Nail”[Title/Abstract] OR “Cephalomedullary Nail”[Title/Abstract] OR “Cephalomedullary Nailing”) AND ("Intertrochanteric Femur Fractur*"[Title/Abstract] OR "Intertrochanteric Fractur*"[Title/Abstract] OR "Hip Fractur*"[Title/Abstract] OR "Proximal Femur Fractur*"[Title/Abstract] OR "Pertrochanteral Fractur*"[Title/Abstract] OR “Pertrochanteric Fractur*”[Title/Abstract]) |
| Embase(1599) | ('intramedullary nailing'/exp OR 'cephalomedullary nail'/exp OR ' Intramedullary Nailing'/exp OR ‘Intramedullary Nail’:ab,ti OR ‘Cephalomedullary Nail’:ab,ti OR ‘Cephalomedullary Nailing’:ab,ti) AND ('femur intertrochanteric fracture'/exp OR 'femur intertrochanteric fracture'/exp OR ‘Intertrochanteric Femur Fractur*’:ab,ti OR ‘Intertrochanteric Fractur*’:ab,ti OR ‘Proximal Femur Fractur*’:ab,ti OR ‘Pertrochanteral Fractur*’:ab,ti OR ‘Pertrochanteric Fractur*’:ab,ti) |
| Web of Science(956) | (TS=("Fracture Fixation, Intramedullary" OR “Intramedullary Nailing” OR “Intramedullary Nail” OR “Cephalomedullary Nail” OR “Cephalomedullary Nailing”)) AND TS=("Intertrochanteric Femur Fractur*" OR "Intertrochanteric Fractur*" OR "Hip Fractur*" OR "Proximal Femur Fractur*" OR "Pertrochanteral Fractur*" OR “Pertrochanteric Fractur*”) |

Supplementary Figure 1 Forest plot of leave-one-out sensitivity analysis for duration of surgery in the short cephalomedullary nails group versus long cephalomedullary nails group.


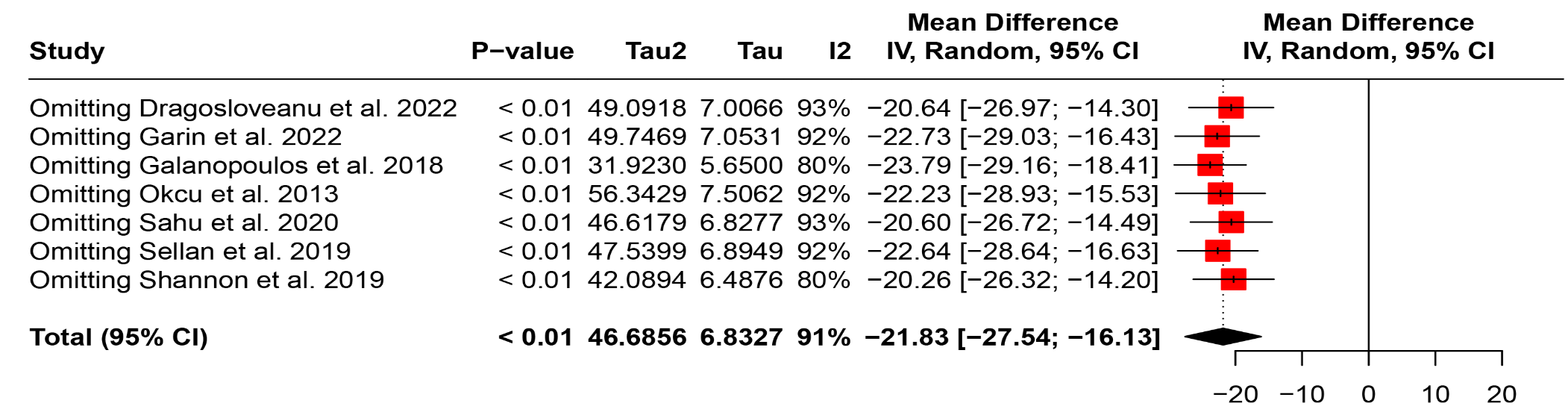


Supplementary Figure 2 Forest plot of leave-one-out sensitivity analysis for lengths of hospital stays in the short cephalomedullary nails group versus long cephalomedullary nails group.


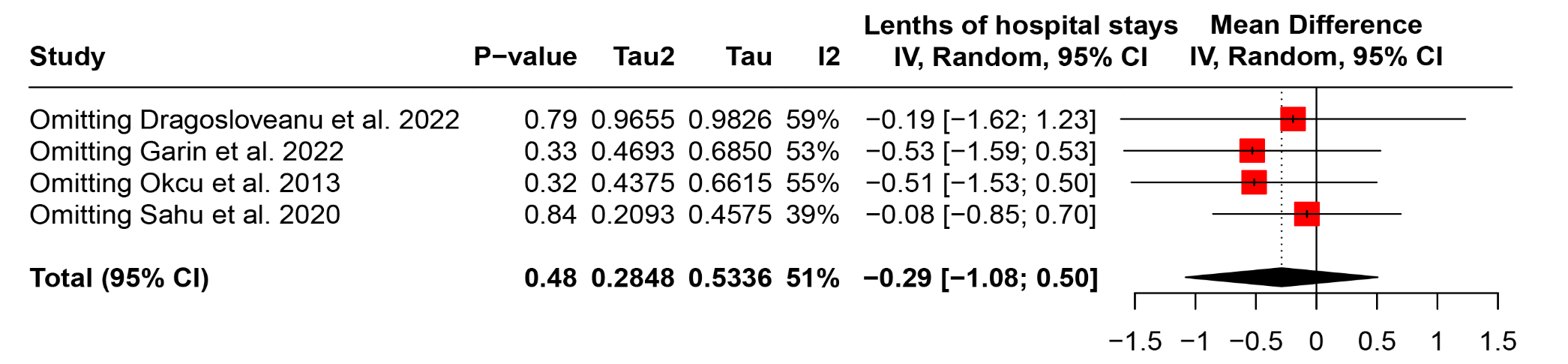


Supplementary Figure 3 Forest plot of leave-one-out sensitivity analysis for Harris hip scores in the short cephalomedullary nails group versus long cephalomedullary nails group.


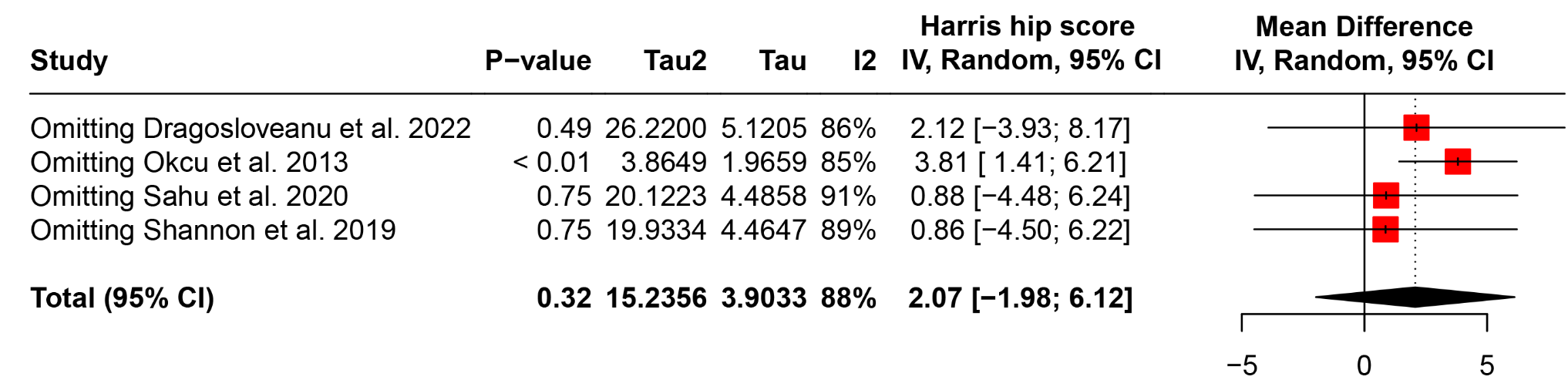

Supplement: S3 File — (DOCX) [file pone.0319758.s005.docx]
