# Supplementary material for: Short versus long cephalomedullary nails for intertrochanteric femur fractures: A meta-analysis of randomized controlled trials
Source: PLoS One. 2025 May 5;20(5):e0319758. doi: 10.1371/journal.pone.0319758 (PMC12052151; doi:10.1371/journal.pone.0319758)
Supplement: Attachment — Submitted filename: Response to Reviewers.docx [file pone.0319758.s007.docx]

Reviewer #1

Overall comment:
Thank you for submitting this comprehensively written manuscript on short versus long cephalomedullary nails for intertrochanteric femur fractures. The methods described are sound and results are described in a clear and concise fashion. It is however, not the first review on the topic, though it does include novel studies not included in previous research. The manuscript does also raise some questions and remarks that might warrant further revision or additional analysis before eventual publication.

Firstly, I do not quite understand why the authors have decided to make surgery time their primary outcome when they state in their aim to compare efficacy and safety, which are arguably much more important. Why not make functional outcomes or complication rates your primary outcome. Or describe in your introduction why you think surgery time is relevant as primary outcome for this analysis. In my opinion this would not be a deciding factor for superiority unless almost all other factors in this consideration were similar. Primary and secondary outcomes should be picked and described in order of importance (quality of life, mortality and functional outcomes -> complications and reoperation -> surgical outcomes and operation characteristics (like blood loss or operation time). A significant difference does not mean that an outcome should become the primary focus of a manuscript.

Response:

Thank you for your feedback. Based on your suggestion, we have revised the manuscript to focus on functional outcomes and mortality as primary outcome measures, reflecting the importance of efficacy and safety in clinical practice. The duration of surgery has now been moved to a secondary outcome.

The revised section now reads:

"The primary outcome measures were functional outcomes, specifically the Harris Hip Score, and mortality within 1 year. Secondary outcomes included complication rates (any adverse events occurring during or after surgery), reoperation rates, the duration of surgery (time from the initial incision to final wound closure), tip-apex distance, intraoperative blood loss, and length of hospital stay."

Additionally, we have updated the order of figures and the presentation of results to reflect this revised prioritization.

Comment 1:
Line 13 (and throughout the manuscript) units (e.g. minutes or centimeters) are missing for many of the outcomes described.

Response:
Thank you for your comment. We agree with your observation and have added the appropriate units (minutes for surgery time, centimeters for tip-apex distance, and milliliters for intraoperative blood loss) throughout the manuscript to ensure clarity and precision in the reported outcomes. The updated paragraph is as follows:

Results: A total of 7 studies with 658 patients were included in this analysis. Durations of surgery were significantly lower in the short CMN group compared to the long CMN group (MD: -21.83 minutes, 95% CI: -27.54 minutes, -16.13 minutes). Furthermore, tip-apex distance was significantly lower in the short CMN group compared to the long CMN group (MD: -0.47 cm, 95% CI: -0.63 cm, -0.31 cm), along with a lower intraoperative blood loss (MD: -136.70 mL, 95% CI: -139.06 mL, -134.34 mL).

Comment 2:
Line 18: No need to mention all were P>0.05, this was already implied by no significant difference.

Response:
Thank you for your suggestion. We have revised the manuscript by removing the reference to P>0.05

Comment 3:
Line 19: It is not common to describe funnel plots results in the abstract, it is also unclear about which outcome the authors are writing here.

Response:
Thank you for your valuable feedback. Regarding your comment on line 19 about the description of funnel plot results, we have removed the related statement as per your suggestion.

Comment 4:
Line 37: Reference (6) is not about trochanteric fractures and therefore does not support the statement made. Please support this statement with adequate literature.

Response:
Thank you for pointing this out. We have replaced reference (6) with a more appropriate source to support the statement. The new reference is:

Reindl, Rudolf et al. “Intramedullary Versus Extramedullary Fixation for Unstable Intertrochanteric Fractures: A Prospective Randomized Controlled Trial.” The Journal of Bone and Joint Surgery, American Volume, vol. 97, no. 23 (2015): 1905-12. doi:10.2106/JBJS.N.01007.

We believe this provides a stronger basis for the statement made.

Comment 5:
Line 37-40: Maybe this statement should be nuanced. Several reviews see only small differences between EMF and IMF, also in unstable fractures (AO type A2, not considering Type A3/reverse obliquity unstable fractures) See recent review by Zeelenberg et al. (PMID: 38175213) and Cochrane review by Lewis et al. (PMID: 35080771)

Response:
Thank you for your valuable feedback. We have revised the relevant section based on your suggestion and cited the proper refences. The updated version is as follows:

"However, several recent reviews suggest that the differences between CMN and DHS are small, particularly in unstable fractures such as AO type A2 fractures, although not including reverse obliquity fractures (8, 9). Despite this, CMN are still increasingly preferred over DHS for the treatment of unstable intertrochanteric fractures, partly due to their ability to provide superior stability in certain fracture patterns (10)."

Comment 6:
Line 41-42: The reasons for change in practice might be more nuance than only (suspected) difference in outcomes and might be highly surgeon or hospital related (Mellema et al, PMID: 33591214)

Response:
Thank you for your insightful comment. We have revised the relevant section to reflect the nuance in the reasons for the shift in practice, as suggested. The updated version is as follows:

“Despite this, CMN are still increasingly preferred over DHS for the treatment of unstable intertrochanteric fractures, partly due to their ability to provide superior stability in certain fracture patterns(10). This change in practice might also be influenced by factors such as surgeon preference, hospital protocols, and regional variations(11).”

Comment 7:
Methods: Did you collect which type of trochanteric fracture was studied in the included studies? Could this influence the outcomes of the meta-analysis? Should a subanalysis be conducted for various fracture types?

Response:
Thank you for your valuable comment. Due to the complexity of the data, we were unable to conduct a subgroup analysis based on different types of trochanteric fractures. However, in response to your suggestion, we have added a new column titled "AO/OTA Fracture" in Table 1. This column includes the distribution of the AO/OTA fracture classifications for both the Short CMN and Long CMN groups. The updated content can be found in the revised Table 1.

| Author | Comparison | AO/OTA Fracture |
| --- | --- | --- |
| Dragosloveanu et al. | Short CMN | 31-A2(80.7%),  31-A3(19.3%) |
|  | Long CMN | 31-A2(77.7%),  31-A3(22.3%) |
| Garín et al. | Short CMN | 31-A2(82%),  31-A3(18%) |
|  | Long CMN | 31-A2(85%),  31-A3(15%) |
| Galanopoulos et al. | Short CMN | 31-A2,31-A3 |
|  | Long CMN | 31-A2,31-A3 |
| Okcu et al. | Short CMN | 31-A3(100%) |
|  | Long CMN | 31-A3(100%) |
| Sahu et al. | Short CMN | 31-A1(33.3%),  31-A2(53.3%),  31-A3(13.3%) |
|  | Long CMN | 31-A1(28.6%)  31-A2(50%)  31-A3(21.4%) |
| Sellan et al. | Short CMN | 31-A1(15.5%),  31-A2 and 31-A3(84.5%) |
|  | Long CMN | 31-A1(15.4%),  31-A2 and 31-A3(84.6%) |
| Shannon et al. | Short CMN | 31-A1(16.2%),  31-A2(76.3%),31-A3(7.5%) |
|  | Long CMN | 31-A1(13.6%),  31-A2(76.1%),31-A3(10.2%) |

Comment 8:
Line 100: Which complications were included in overall complication rate? What complications were included in this measure and were they different for the included studies?

Response:
We have added more detailed information regarding the complications included in the overall complication rate in the results section of the manuscript.

The complications reported in the studies were as follows: Dragosloveanu et al. (2022) identified screw cut-out and local postoperative hematoma; Garín et al. (2022) noted mechanical issues such as blade lateral migration, cut-through, malrotation, pin fatigue/fracture, and non-union, along with infections and other general complications; Galanopoulos et al. (2018) reported the z-effect phenomenon and periprosthetic fractures; Okcu et al. (2013) observed superficial and deep surgical site infections; and Shannon et al. (2019) mainly reported screw cut-out and deep surgical site infection.

Comment 9:
Was tip-apex distance the only surgical outcome that was studied or did the studies also show data on other operation quality indicators?

Response:
Thank you for your comment. Tip-apex distance was not the only surgical outcome studied. We also evaluated several other surgical quality outcomes(such as Harris Hip Score).

However, tip-apex distance is an important stability indicator, and to the best of our knowledge, it has not been included in previous meta-analyses. In our study, we introduced this measure as a new surgical outcome to assess the differences between the Short CMN and Long CMN groups.

Comment 10:
Line 108: Were all studies sufficiently homogeneous in terms of included patient characteristics and/or fracture types to warrant usage of a fixed effects model?

Response:
Thank you for your comment. In response to your concern, we reviewed the fracture types across the included studies. Upon re-examination, we found that the majority of patients were categorized into 31-A2 and 31-A3 fractures(We have added this information in Table 1). However, we acknowledge that the fracture types were not entirely homogeneous across all studies, which is a limitation of any meta-analysis.

Regarding the choice of statistical model, we selected the fixed-effects model based primarily on the statistical homogeneity of the data. Specifically, when the Cochran’s Q test yielded a P-value less than 0.10 and the I^2^ statistic was less than 50%, we opted for the fixed-effects model[1]. This approach ensures that the model used was appropriate based on the observed data heterogeneity.

We hope this clarifies the rationale behind our choice of statistical model.

[1] Yang K, Kwan H Y, Yu Z, et al. Model selection between the fixed-effects model and the random-effects model in meta-analysis[J]. Statistics and its Interface, 2020, 13(4): 501-510.

Comment 11:
Line 121: Was it not possible to translate non-English articles to be able to include their data?

Response:
Thank you for your comment. Regarding your question about non-English articles, we initially set clear inclusion and exclusion criteria that specifically excluded studies published in languages other than English. This decision was made for several reasons: First, non-English literature can be difficult for readers to access and interpret, which could limit the broader impact and utility of the findings. Second, many studies published in languages such as Spanish or Chinese may have lower methodological rigor or may not meet the same quality standards as those published in English.

As such, we only included studies that had full-text articles available in English. We have emphasized this in our inclusion and exclusion criteria: "Additionally, studies published in languages other than English were excluded due to accessibility issues for readers and concerns regarding the consistency of study quality across different languages."

Comment 12:
Line 137 (and throughout result section): I would not say quantitative results as you do not describe any qualitative results either. Just using the outcome compared as a title is sufficient.

Response:
Thank you for your comment. We will revise the manuscript accordingly. Instead of using the term "quantitative results," we will refer to the outcomes by their specific titles, such as "Harris Hip Score," "mortality within 1 year," "complication rates," etc., to maintain clarity and precision in describing the findings. We have revised in the manuscript.

Comment 13:
Line 139: Please include the appropriate unit when describing outcomes (minutes or seconds or hours/mm cm meter)

Response:
Thank you for your comment. We have added the appropriate units to the outcomes where applicable. This revision has been made throughout the results section.

Comment 14:
Line 145: While the Eggers statistic did was non-significant, about 50% of the included studies fell outside the funnel plot. Please comment on whether this may have had influence on the generalizability of these results.

Response:
Thank you for your comment. We have revised the paragraph to address your concern. Here is the updated version:

“Additionally, funnel plot analysis and Egger's test showed no significant publication bias (P = 0.07) (Figure 8). However, it is worth noting that approximately 50% of the included studies fell outside the funnel plot, which may raise concerns about the potential influence on the generalizability of these results.”

Comment 15:
Discussion: Please first list your main findings and then place them in a scientific context. The first paragraph is more or less a repetition of your introduction and does not explain the relevance of the findings of your analysis.

Response:
Thank you for your valuable feedback. We have revised the first paragraph of the discussion section as suggested. The updated version now clearly presents our main findings and provides their scientific context. Below is the modified text:

Revised First Paragraph:

In the treatment of IFFs, intramedullary nails are a widely accepted and effective option, with both long and short CMN offering distinct advantages(27). Our meta-analysis demonstrated that short CMN are associated with significantly shorter durations of surgery, lower tip-apex distance, and reduced intraoperative blood loss compared to long CMN in the fixation of IFFs. These findings may be attributed to the technical differences between the two types of nails. Short CMN are quicker to insert due to their reduced length, which requires less reaming of the femoral canal and simpler distal locking procedures(15, 16, 18). The shorter length also minimizes the distance the implant must traverse, leading to a reduced tip-apex distance, which is a crucial factor in achieving optimal screw placement within the femoral head(18). Furthermore, the less invasive nature of the procedure with short CMN results in lower intraoperative blood loss, likely due to decreased soft tissue and bone disruption during insertion(15, 16, 18).

We hope this revised version addresses your concerns and improves the clarity of our findings.

Comment 16:
Line 206-209: Please do not repeat extensive results (of many outcomes including CI's) in your discussion if not necessary, unless comparing results to those in other research.

Response:
Thank you for your comment. We have revised the discussion section by removing the extensive results and confidence intervals, as suggested. Only key points are now presented.

Comment 17:
Line 245: Despite the difference between groups, was the average tip apex distance not <25mm in both populations, suggesting adequate implant placement? Is this difference then a relevant outcome?

Response:
Thank you for your valuable comment regarding the tip-apex distance(TAD). We agree that both groups in our meta-analysis had an average TAD<25mm, which generally suggests adequate implant placement and is associated with favorable clinical outcomes. However, the statistically significant difference in TAD between the short and long CMN groups still warrants consideration.

While the absolute difference in TAD between the two groups may be small, it is important to note that even minor variations in TAD can have clinical relevance, particularly in complex fractures. In cases where the bone quality is compromised or the fracture pattern is unstable, small differences in screw placement may influence the overall stability of the implant and potentially affect long-term outcomes, such as the risk of screw cut-out or implant failure[1]. Additionally, achieving an optimal TAD is crucial for minimizing complications, even if the measurements are within an acceptable range[2]. Thus, we believe that this difference, albeit small, may still provide valuable insight into the nuances of surgical outcomes between the two groups.

[1]Reindl R, Harvey E J, Berry G K, et al. Intramedullary versus extramedullary fixation for unstable intertrochanteric fractures: a prospective randomized controlled trial[J]. JBJS, 2015, 97(23): 1905-1912.

[2] Caruso G, Corradi N, Caldaria A, et al. New tip-apex distance and calcar-referenced tip-apex distance cut-offs may be the best predictors for cut-out risk after intramedullary fixation of proximal femur fractures[J]. Scientific reports, 2022, 12(1): 357.

Comment 18:
Discussion: Please comment on the clinical relevance of the differences you found. Is the magnitude of surgery duration or blood loss great enough to change clinical practice? How do difference in costs influence these decisions?

Response:
Thank you for your insightful comments. In response to your suggestion, we have revised the discussion to address the clinical relevance of the differences observed in our study.

The revised paragraphs now updated:

Our results demonstrate that short CMN are associated with shorter surgery times, reduced tip-apex distance, and lower intraoperative blood loss compared to long CMN, suggesting that short CMN may be a better option in terms of efficacy. However, since the higher heterogeneity in some of our results and the relatively small number of included RCTs, further researches are needed to obtain more robust conclusions. In addition, while these differences are statistically significant, the clinical relevance of these findings depends on the context. Although shorter surgery duration and reduced blood loss may lead to faster recovery and lower risk of complications, it is important to consider whether these benefits are large enough to influence clinical practice. In many cases, the absolute difference in surgery duration (approximately 22 minutes) and blood loss (reduced by 136.7 mL) might be considered modest in the broader context of the patient's overall treatment and recovery(5, 15). Additionally, the choice between short and long CMN should not only rely on these intraoperative factors but also on other considerations such as fracture type, patient comorbidities, and cost(12, 26). The choice between short and long CMN in clinical practice should be tailored to the individual patient’s condition, considering the specific advantages and potential risks of each option.

Comment 19:
Line 257: Was is the number of trials that was low or the number of included patients?

Response:
Thank you for your comment. To clarify, the issue regarding sample size bias in our analysis stems primarily from the limited number of randomized controlled trials included in the study (7 trials), rather than the number of patients.

The updated sentence now reads:

Secondly, the relatively small number of randomized controlled trials (only 7 trials) included in this analysis, may introduce a small sample size bias.

Once again, thank you for your work!!

#Reviewer 2

Comment 1:
Line 21: „Future larger sample size randomized controlled trials are needed to validate the current conclusion." Delete that from the abstract conclusions.

Line 26: „Intertrochanteric femur fractures (IFFs) are a common type of hip fracture, …" No. They are not really hip fractures. They are a common type of proximal femur fractures.

Response:

Thank you for your insightful comment. As per your suggestion, we have removed the phrase "Future larger sample size randomized controlled trials are needed to validate the current conclusion" from the conclusion section of the abstract to avoid unnecessary repetition. In addition, we have revised the sentence to accurately reflect that intertrochanteric femur fractures (IFFs) are a type of proximal femur fracture, not specifically hip fractures.

“Intertrochanteric femur fractures (IFFs) are a common type of proximal femur fracture, particularly prevalent among the elderly population due to factors such as osteoporosis and decreased bone density”

Comment 2:

No study protocol registration in PROSPERO or elsewhere?

Response:
Thank you for your comment. We would like to inform you that we have indeed registered the protocol for this meta-analysis with PROSPERO. The registration identifier is CRD42024616788.

Comment 3:Why did not you search CNKI or other Chinese databases? There is so many research going on in China.

Response:
Thank you for your valuable comment. Regarding your question about non-English articles, we initially set clear inclusion and exclusion criteria that specifically excluded studies published in languages other than English. This decision was made for several reasons:

- Accessibility and Interpretation: Non-English literature can be difficult for readers to access and interpret, which could limit the broader impact and utility of the findings.
- Methodological Quality: Studies published in languages such as Spanish or Chinese may not always meet the same methodological rigor or quality standards as those published in English.

As such, we decided to include only studies that had full-text articles available in English. This has been emphasized in our inclusion and exclusion criteria: "Additionally, studies published in languages other than English were excluded due to accessibility issues for readers and concerns regarding the consistency of study quality across different languages."

Comment 4:Please give an exact definition of short and long nails. How many cm?

Response:
Thank you for your suggestion. In response to your request for a clear definition of short and long intramedullary nails, we have specified the exact lengths in the revised manuscript.

1.Long CMN: Length ranging from 240 to 460 mm.

2.Short CMN: Length ranging from 170 to 200 mm.

Comment 5:
You should provide information what the best tip apex distance is in the literature. What does a reduced tip apex distance mean? Is it better or worse according to your findings under consideration of the best tip apex distance described in the literature. Furthermore, report the overall mean tip apex distance of all short nail and all long nail studies. Are the values reported in the forest plot presented in mm?

Response:
Thank you for your insightful comment. We appreciate the opportunity to clarify the clinical significance of the tip-apex distance.

**Unit of Measurement:** The values reported in the forest plot for TAD are indeed in millimeters (mm). We have updated this in the results section to avoid any ambiguity.

**Best Tip-Apex Distance in the Literature:** According to the literature, a tip-apex distance of less than 25 mm is generally considered optimal for implant placement in intertrochanteric fractures. This threshold is associated with a reduced risk of screw cut-out and better overall outcomes.

**Interpretation of Reduced TAD:** In our findings, the shorter TAD observed in the short CMN group (mean difference: -0.47 mm, 95% CI: -0.63 mm to -0.31 mm) suggests slightly better implant positioning compared to the long CMN group. While the absolute difference is small, the reduction in TAD could indicate a more precise placement, which may contribute to improved stability. However, both groups had TAD values well below the recommended 25 mm threshold, suggesting that implant placement was adequate in both groups.

**Overall Mean TAD:** The overall mean TAD across all included studies was as follows:

Short CMN: Weighted mean TAD = 18.3 mm.

Long CMN: Weighted mean TAD = 19.0 mm. We have added this to the revised manuscript:

Short CMN showed a weighted mean TAD of 18.3 mm, while long CMN demonstrated a weighted mean TAD of 19.0 mm. The tip-apex distance was significantly lower in the short CMN group compared to the long CMN group (MD: -0.47 cm, 95% CI: -0.63 cm, -0.31 cm) (Figure 10).

Comment 6:

Then, you should discuss WHY there is a shorter tip apex distance in short nails, because the reason does not seem obvious. I cannot think of any reason why any operator would tend to proceed different according to shorter or longer nails in terms of the tip apex distance.

Response:

Thank you for your valuable comment. Regarding the shorter tip-apex distance (TAD) observed with short nails, we have provided further clarification. We believe that the shorter length of short CMN reduces the distance the implant must traverse, which can simplify the alignment process during surgery and allow surgeons to place the lag screw more precisely within the femoral head. This simplified process may result in a slightly reduced TAD, as surgeons may have greater control over the screw trajectory and final positioning when using shorter implants.

We have revised the relevant paragraphs to further explained:

The shorter length of short CMN minimizes the distance the implant must traverse, potentially simplifying the alignment process during surgery and allowing for more precise placement of the lag screw within the femoral head(18, 26). This precision may result in a reduced tip-apex distance, as the surgeon may have greater control over the screw trajectory and final positioning when dealing with shorter implants(12, 15). Furthermore, the reduced length of short nails may decrease procedural complexity in aligning the distal nail, allowing surgeons to focus more on achieving optimal screw placement, which is a crucial factor in minimizing complications such as screw cut-out(18).

Comment 7:

Line 261: Here you use „surgery times" instead of the before mentioned „duration of surgery". Unify your terms. The „reduced tip apex distance" is an interesting finding, but it is hard to interprete because of the missing information I mentioned before.

All in all, this is a valuable meta-analysis. Statistics and methods are fine. Good work. I am very interested in reading your changes.

Response:
Thank you very much for your thoughtful comments and your positive feedback on the statistical analysis and methods. In response to your comment regarding the terminology, we have carefully reviewed and updated the manuscript to ensure consistency. We have unified all manuscript from "surgery times" to "duration of surgery," as suggested.

We sincerely appreciate your interest in our changes, and we believe these revisions will enhance the clarity and consistency of our findings. Once again, thank you for your work!!
