# Supplementary material for: Short versus long cephalomedullary nails for intertrochanteric femur fractures: A meta-analysis of randomized controlled trials
Source: PLoS One. 2025 May 5;20(5):e0319758. doi: 10.1371/journal.pone.0319758 (PMC12052151; doi:10.1371/journal.pone.0319758)
Supplement: Attachment — Submitted filename: Response_to_Reviewers_auresp_2.docx [file pone.0319758.s008.docx]

Reviewer #1

Comment 1:
Maybe the authors could also follow the same order of results in the manuscript abstract as in the methods/results. Now the result section of the abstract still starts with the significant results and not with function or complications.

Response:
Thank you for this helpful suggestion. We have revised the results section of the abstract to follow the same order as the results section in the manuscript (3.3–3.11). Specifically, we now present the findings related to Harris hip score, 1-year mortality, overall complications, and reoperation rates before describing the statistically significant differences in operation duration, intraoperative blood loss, and tip-apex distance. The revised abstract result section is as follows:

"A total of 7 studies with 658 patients were included in this analysis. There was no significant difference between the short CMN group and the long CMN group in Harris hip score, mortality within 1-year, overall complication rates, or reoperation rates. However, durations of surgery were significantly lower in the short CMN group compared to the long CMN group (MD: -21.83 minutes, 95% CI: -27.54 minutes, -16.13 minutes), along with significantly lower intraoperative blood loss (MD: -136.70 mL, 95% CI: -139.06 mL, -134.34 mL) and tip-apex distance (MD: -0.47 cm, 95% CI: -0.63 cm, -0.31 cm). There was also no significant difference in peri-implant fracture or lengths of hospital stays."

We hope this revised structure addresses your concern and improves the clarity of the abstract.

Comment 2:
Please include no differences in functional outcomes, overall complications, and reoperation in the conclusion of your abstract and conclusion section. This information could also be given a more prominent place in the first paragraph of the discussion section. It is important to keep in mind that while TAD, operation time, and bloodloss were significantly different, the absolute differences were small and the clinical relevance of these differences are debatable and likely small. The two groups showed no differences in other (arguably more important) outcomes. Your discussion/conclusions should reflect this.

Response:
Thank you for this insightful suggestion. We have made the following revisions to address your comment:

**Conclusions**:
We have revised the conclusion section to include the lack of significant differences in functional outcomes, overall complication rates, reoperation rates, and mortality within one year. The updated conclusion now reads as follows:
"Short CMN are associated with shorter duration of surgery, reduced tip-apex distance, and lower intraoperative blood loss compared to long CMN for the fixation of IFFs. However, there were no significant differences in functional outcomes, overall complication rates, reoperation rates, mortality within one year, peri-implant fracture, or lengths of hospital stays."

**Discussion**:
To reflect the limited clinical relevance of the statistically significant differences, we added the following sentence to the first paragraph of the discussion section:
"These differences were statistically significant but small in absolute terms."
